# 

**Published:** 1845-02

**Authors:** 


### CONTENTS

OF THE

### MEDICAL EXAMINER.

NEW SERIES—NO. II.—FEBRUARY, 1845.

### ORIGINAL COMMUNICATIONS.

Case of Racemiferous Hydatids of the Uterus. Reported by J. K. Mitchell, M. D., - - - - - - - - - - - 73
On the Prevailing Influenza. By James Bryan, M. D., - - - - 76
Treatment, - - - - - - - - - - - 77
On Strictures of the Rectum. By Thomas D. Mütter, M. D., Professor of Surgery in Jefferson Medical College, Philadelphia, - - - - 77
Of Simple or Non-Malignant Stricture, - - - - - - - 78
Spasmodic Stricture, - - - - - - - - - - 78
Permanent Stricture, - - - - - - - - - - 80

### CLINICAL LECTURES AND REPORTS.

PHILADELPHIA HOSPITAL,

*Saturday, December* 14, 1844.

Clinic of Professor Dunglison.
Intermittent Fever, - - - - - - - - - - - - - 90
Jerking Respiration, - - - - - - - - - - - - - 96
Asthma, - - - - - - - - - - - - - - - - - 96
Pathological Specimens, - - - - - - - - - - - - 98

### BIBLIOGRAPHICAL NOTICES.

A Practical Treatise on the Diseases of the Respiratory Organs; including Diseases of the Larynx, Trachea, Lungs, and Pleura. By Charles J. B. Williams, M. D., F. R. S., Fellow of the Royal College of Physicians, Professor of the Principles and Practice of Medicine, and of Clinical Medicine, and First Physician to the Hospital, University College, London, &c. With numerous Additions and Notes. By Meredith Clymer, M. D., Physician to the Philadelphia Hospital, Fellow of the College of Physicians, &c. &c. With Woodcut Illustrations. 8vo. pp. 508. - - - - - - - 103

Medical Lexicon or Modern Terminology; being a complete Vocabulary of Definitions, including all the technical terms employed by Writers and Teachers of Medical Science at the present day, and comprising several hundreds of words not found in any other Dictionary. Designed for the use of Students and Practitioners. By David Meredith Reese, A. M., M. D., of New York. Late Professor of the Institutes of Medicine and Surgery and Medical Jurisprudence in the Washington University of Baltimore, Editor of Cooper's Surgical Dictionary, &c. 24mo. pp. 229. - - - - - - 106

Observations on the Structure and Diseases of the Testis; with numerous plates. By Sir Astley Cooper, Bart., F. R. S., Sergeant Surgeon to the Queen, Consulting Surgeon to Guy's Hospital. From the Second London Edition. Edited by Bransby B. Cooper, F. R. S., Surgeon to Guy's Hospital, Lecturer on Anatomy, - - - - 109

The Anatomy of the Thymus Gland, with numerous plates. By Sir Astley Cooper, etc. etc. etc. Royal Octavo. - - - - 109

American Medical Biography; or, Memoirs of Eminent Physicians, embracing principally those who have died since the publication of Dr. Thacher's work on the same subject. With Engravings. By Stephen W. Williams, M. D., Late Professor and Lecturer upon Materia Medica, Medical Jurisprudence, etc., in the Medical College of Berkshire, Mass., etc. etc. 8vo. pp. 664, - - - - - 110

Medical Topography of Brazil and Uruguay; with Incidental Remarks. By G. R. B. Horner, M. D., Surgeon U. S. Navy, Honorary Member of the Philadelphia Medical Society, and Corresponding member of the National Institute at Washington. 8vo. pp. 296, - - 111

The Medical Student's Vade Mecum, containing examinations upon Anatomy, Chemistry, Materia Medica, Surgery, Practice of Medicine, Obstetrics and Poisons. Adapted to the use of Medical Students generally. By George Mendenhall, M. D., &c. One volume, 12mo. pp. 328, - - - - - - - - - - - 112

The first lines of the Theory and Practice of Surgery; including the principal operations. By Samuel Cooper, Senior Surgeon to University College Hospital, and Professor of Surgery in the same College, etc. With notes and additions, by Willard Parker, M. D., Professor of Surgery in the College of Physicians and Surgeons in the University of the State of New York, etc. etc. Fourth American, from the Seventh London Edition. In two volumes, 8vo., - - - 113

### EDITORIAL.

American Journal of Insanity, - - - - - - - - 114

Southern Medical and Surgical Journal, - - - - - - 115

Murder—Quack Medicines, etc., - - - - - - - 115

British and Foreign Medical Review, - - - - - - - 116

### RECORD OF MEDICAL SCIENCE.

Medical Institution of Yale College, - - - - - - - 116

A Report on the Yellow Fever that recently prevailed at Woodville, Miss. Read in French and English before the Medico-Chirurgical Society of Louisiana, on the 2d October, 1844. By Doctors C. De Valetti and Thomas M. Logan, a committee delegated for the purpose, - 117

Report of the trial of Abner Rogers, Jr., indicted for the Murder of Charles Lincoln, Jr., late Warden of the Massachusetts State Prison; before the Supreme Judicial Court of Massachusetts; holden at Boston, on Tuesday, January 30th, 1844. By George Tyler Bigelow and George Bemis, Esqs., Counsels for the defendant, - - - 124
Nervous Fluid, - - - - - - - - - - - - 135
Rhode Island Lunatic Asylum, - - - - - - - - 135
First Lunatic Asylum in the United States, - - - - - - 135
Exemption of the Cherokee Indians and Africans from Insanity, - - 135
Fractures of the Cervix Scapulæ, - - - - - - - - 136
Perforation of the Ileum, - - - - - - - - - - - 136
Fungus Hæmatodes, - - - - - - - - - - - - - 136

